# Fast and accurate gene regulatory network inference by normalized least squares regression

**DOI:** 10.1093/bioinformatics/btac103

**Published:** 2022-02-17

**Authors:** Thomas Hillerton, Deniz Seçilmiş, Sven Nelander, Erik L L Sonnhammer

**Affiliations:** Department of Biochemistry and Biophysics, Stockholm University, Science for Life Laboratory, 17121 Solna, Sweden; Department of Biochemistry and Biophysics, Stockholm University, Science for Life Laboratory, 17121 Solna, Sweden; Science for Life Laboratory, Department of Immunology, Genetics and Pathology, Uppsala University, 75185 Uppsala, Sweden; Department of Biochemistry and Biophysics, Stockholm University, Science for Life Laboratory, 17121 Solna, Sweden

## Abstract

**Motivation:**

Inferring an accurate gene regulatory network (GRN) has long been a key goal in the field of systems biology. To do this, it is important to find a suitable balance between the maximum number of true positive and the minimum number of false-positive interactions. Another key feature is that the inference method can handle the large size of modern experimental data, meaning the method needs to be both fast and accurate. The Least Squares Cut-Off (LSCO) method can fulfill both these criteria, however as it is based on least squares it is vulnerable to known issues of amplifying extreme values, small or large. In GRN this manifests itself with genes that are erroneously hyper-connected to a large fraction of all genes due to extremely low value fold changes.

**Results:**

We developed a GRN inference method called Least Squares Cut-Off with Normalization (LSCON) that tackles this problem. LSCON extends the LSCO algorithm by regularization to avoid hyper-connected genes and thereby reduce false positives. The regularization used is based on normalization, which removes effects of extreme values on the fit. We benchmarked LSCON and compared it to Genie3, LASSO, LSCO and Ridge regression, in terms of accuracy, speed and tendency to predict hyper-connected genes. The results show that LSCON achieves better or equal accuracy compared to LASSO, the best existing method, especially for data with extreme values. Thanks to the speed of least squares regression, LSCON does this an order of magnitude faster than LASSO.

**Availability and implementation:**

Data: https://bitbucket.org/sonnhammergrni/lscon; Code: https://bitbucket.org/sonnhammergrni/genespider.

**Supplementary information:**

Supplementary data are available at *Bioinformatics* online.

## 1 Introduction

Inferring accurate gene regulatory networks (GRNs) is a key goal in the field of systems biology (Kitano, 2002). However, gene regulatory network inference (GRNI) is a highly complex problem and currently at least two factors severely limit the predictive ability of GRNI methods. First, there is an inherent complexity in biological systems making it hard to find accurate models. Second, and perhaps even more central, is the current lack of knowledge of expected properties in a biological GRN, and how patterns of gene interactions coalesce on a large scale. Today, only a relatively small number of gene regulatory interactions have been experimentally validated and it is still largely unclear what properties a true biological GRN should have (Banf and Rhee, 2017). Until more knowledge is available for true GRNs it is important to construct inference methods that account for this shortcoming by being adequately conservative in their predictions. From the point of view of a biologist that wants to pursue follow-up experiments, it is often more important to minimize the number of false interactions than finding all possible true interactions. A further challenge for GRNI is ensuring that the inference method can be used at the increasing scale of gene expression data available, something which has become especially important in recent years (Sanguinetti and Huynh-Thu, 2018; Subramanian et al., 2017) . A method that encapsulates both scalability and high accuracy is the Least Squares Cut-Off (LSCO) method (Tjärnberg *et al.*, 2013). LSCO relies on a computationally low complexity algorithm based on least squares fitting. It posits that by perturbing each component in a system according to a known experimental design matrix, and measuring the system-wide gene expression effect of said perturbations at steady-state, a high-quality GRN can be obtained through least squares-based linear regression (Tjärnberg *et al.*, 2013).

LSCO is however affected by the inherent limitations of ordinary least squares. One such limitation, which is the focus of this study, is a vulnerability to extreme values in data (Anscombe, 1973). The issue arises from ordinary least squares using inversion of the observed variable to fit the model to the data. In this inversion, values that are considerably larger or smaller than the average will be massively amplified or reduced, giving a false sense of importance or unimportance in the fitted model. In LSCO, this results in a large number of incorrect highly weighted links, especially for small values close to zero.

## 2 Materials and methods

### 2.1 Data used

We used both synthetic and real data for this study. To measure correctness, simulated data generated with the GeneSPIDER tool were used as it ensures that there is a complete, known true GRN for comparison (Tjärnberg *et al.*, 2017). To test prediction capability over varying difficulties, data were generated with low to high noise, using signal-to-noise ratio (SNR) steps of 1, 0.1, 0.01 and 0.001. SNR 1 corresponds to almost no noise and at SNR 0.001 almost all signal is masked by the noise. To show that Least Squares Cut-Off with Normalization (LSCON) works on other data, we also used simulated data from the GeneNetWeaver (GNW) tool (Schaffter *et al.*, 2011). As GNW does not allow for varying the noise only high noise datasets were used here. For GeneSPIDER, two types of data were created, one with properties that are susceptible to induce hubs, here called hub-prone data. The other type does not have properties that are prone to induce hubs, here called balanced data. For GNW, only balanced data were used as the aim here is to ensure that the initially observed trends were not caused by the GeneSPIDER simulation model.

For both simulation models, the data were used as fold changes. For GeneSPIDER these are directly simulated, whereas for GNW they were calculated as log2 of the ratio between the simulated and wild-type expression levels. Further details on data generation and properties of the generated data are available in Supplementary Note S1. To complement the simulated data, experimental data from two human cell lines, A375 and A549, from the LINCS L1000 (Subramanian *et al.*, 2017) were used. The cells are derived from human melanoma and human lung carcinoma cells, respectively. The L1000 datasets had an SNR comparable to that of the synthetic data, with an SNR value of 0.0019 for A375 and 0.0017 for A549. The condition numbers (the largest singular value divided by the smallest singular value) were 824 and 665, respectively, which lies in between the hub-prone and balanced data (Supplementary Table S1). These particular datasets were selected partly as this is where the megahub problem initially was encountered, but more importantly to ensure that the issue could be solved both in simulated data and real data. The L1000 data used here are experimental gene knock down data where each gene measured has been knocked down one by one using shRNA in three experimental replicates with multiple shRNAs for most genes. The effect was then measured at 72–96 h after perturbation using the L1000 luminex bead system (Peck *et al.*, 2006). The data were downloaded in the Level 3 format provided at GEO: GSE92742. Before applying any GRNi method, the data were further processed by removing any shRNA not present in all replicates and averaging the effect of the remaining shRNAs for each gene, to compensate for any off-target effects or unknown differences in knock down strength.

SNR for both synthetic and experimental data was calculated using the equation:

(1) $$\mathrm{SNR}=\frac{\Sigma_{\min}\left(Y\right)}{\sqrt{\chi^{2}(\alpha ,N,\;M)\lambda }},$$

where Σ_min_ is the smallest singular value, *Y* the measured fold change in gene expression, $\chi^{-2}\left(\alpha ,NM\right)$ the chi-square distribution with N × M degrees of freedom and *λ* the normally distributed variance of the noise. *N* is here the number of studied genes and *M* the number of experiments performed. Note that to obtain regularly spaced SNR levels for the data simulated by GeneSpider, *Y* was used without any noise for the data simulation.

### 2.2 Inducing infinitesimal values in simulated data

In order to guarantee that the simulated data contained values close to zero with the potential to cause megahubs, an algorithm was developed for adding noise to random positions in the data. The algorithm takes a generated synthetic dataset and returns a modified response matrix. The modification affects 15% of the genes or a minimum of 10 genes. For these genes, randomly selected, all expression fold change values are divided by a random value such that each fold change value is rescaled according to:

(2) $$C_{i}=\frac{G_{i}}{R\in \left[0.5N,\;0.5N+250\right]},$$

where *G* is a vector of fold change values, *C* a vector of rescaled fold change values, *N* the total number of elements in *G*, and *R* a random number in the range 0.5*N* to 0.5*N* + 250. *R* is stochastic to ensure some variation in how strong the effect of the infinitesimal values has on the system.

### 2.3 Least Squares Cut-Off with Normalization

**Fig. 1.**
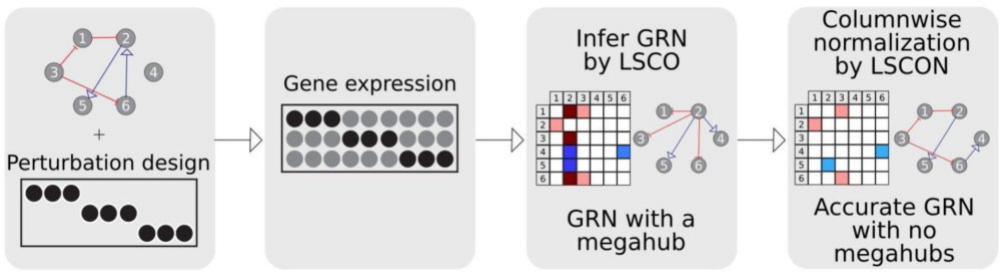
LSCON workflow. LSCON works by performing a least-squares fit between the gene expression data and a perturbation matrix and then applying a column-wise normalization to the resulting GRN matrix to reduce extreme values

LSCON builds on the previously published method LSCO but adds a normalization step of the network prediction to scale all gene interactions to a similar scale (Fig. 1), to limit the effect of extreme values in the input data. The idea to normalize the coefficients of a least squares regression builds on previous work on standardized regression that showed that rescaling coefficients is allowed as it maintains the relative importance among them (Bring, 1994). The rescaling is performed column-wise such that the absolute sum of each column is the same across the whole data, thereby reducing outlier effects. Outliers and extreme values are known problems in ordinary least squares fitting (Bronson and Costa, 2021), the underlying method that both LSCO and LSCON build on. The effect in the fit comes from the inversion used by least squares to fit its model. This inversion leads to amplification or reduction of extreme values, giving them a false importance based on the faulty score. For inferred GRNs, this effect is observed in data where a gene has almost no change in gene expression, causing all the fold change values to be extremely close to 0 (<0.001). These small values are then inverted in the least squares fit, giving some genes a large number of very strong interactions that are not real. By normalizing the predicted network, we can effectively reduce these extreme predictions back into more realistic values while adding very little overhead to the computationally efficient LSCO method. The normalization is done using the equation:

(3) $$X_{ij}=\frac{A_{ij}}{\sum_{}^{}\left(\left|A_{j}\right|\right)N},$$

where *N* is the number of genes, *A* is the original predicted GRN, *X* the normalized GRN, *j* is a regulator gene in column *j* in the range 1 to *N*, and *i* is a target gene in row *i* in the range 1 to *N*. This is done for each value in the network, ensuring that the total column sum is equalized across the network.

### 2.4 Benchmarking

To benchmark LSCON against GRNI methods other than LSCO, we compared it to a set of methods that have previously been shown to accurately predict GRNs. The methods are LSCO, LASSO, (Tjärnberg *et al.*, 2013, 2015), ridge regression (Friedman *et al.*, 2010) with cut-off (RidgeCO) and Genie3 (Huynh-Thu *et al.*, 2010); note that the Matlab version of Genie3 was used here. All methods were tested both on hub-prone data and balanced data. All of these methods are available in the GeneSPIDER package (Tjärnberg *et al.*, 2017) in Matlab, allowing for easy use and comparison to LSCON. All methods have been set up to generate a set of GRNs over a set of sparsities. Each method produces a full GRN, i.e. a network with all possible edges included. From this, a series of 30 GRNs are created by stepwise reducing the number of total edges from all to zero predicted edges based on the score of the edge. Similar sparsity intervals were used for different methods to ensure a fair comparison. When evaluating the benchmark, the inferred GRN with a sparsity closest to the true GRN’s sparsity was used throughout this work. All of the methods used for this benchmark are made available in the GeneSPIDER repository. A number of popular methods including TIGRESS, and ARACNe were not included in this study as Genie3 has been shown to outperform them in the DREAM5 challenge ((Marbach *et al.*, 2012).

### 2.5 Measuring performance

For this study three different performance measurements were used. Run time was measured using Matlab's CPU time function (Matlab, cputime). For timing, all methods were run on an Intel Xeon E5-2690v3 CPU with 48 cores and 512 GB of RAM memory. Area under the precision-recall (AUPR) curve was used to determine the overall correctness of a method based on precision and recall. Recall measures how many of the links in the simulated true GRN a method can correctly predict and precision how many of the predicted links over all predictions are actually true (Prill *et al.*, 2010). The use of AUPR allows for a correctness measurement that accounts for both true positives and false positives while not accounting for true negatives, i.e. no link. When working on sparse data like GRNs, not accounting for true negatives offers an advantage that the relatively high number of zeros will not artificially increase the accuracy. For capturing correctness in each different sparsity for the generated GRNs, the F1 score was instead used as this one allows for a single network to be compared with the true. To test for statistical significance in the correctness measurements the R programming language’s (R Core Team) rank-sum test function was used (R Core Team, wilcox.test). Finally, as a key question in this study, a method's ability to avoid amplifying infinitesimal values was tested by looking at the maximum out-degree. Out-degree was used rather than in-degree or total degree as it was reasoned that removing false regulators, especially with this large number of regulations, was more relevant than a gene under control of too many regulators. The reason is that hub regulators are more prone to be used to draw biological conclusions from.

## 3 Results

The LSCON algorithm was evaluated by measuring performance in three categories: (i) ability to avoid megahubs, (ii) similarity between the predicted GRN and the true GRN and (iii) execution time. LSCON’s performance was compared to that of established GRNI methods: LASSO, Genie3, LSCO and ridge regression (RidgeCO). As an initial test to verify that LSCON can fill the role of LSCO all methods were run on balanced data without infinitesimal values. Here all methods performed well with LSCON, LSCO, RidgeCO and LASSO with an AUPR above 0.8 on SNR 1.0. Genie3 had a lower AUPRs of about 0.3 (Supplementary Fig. S1). LSCO and LSCON here performed equally well (rank-sum test *P*-value 1). For further details on these results see Supplementary Figure S1. Once the identical results were confirmed all further testing was carried out on hub-prone data, data with small values of <0.0001, as this is the key focus of this study. Note that Genie3 was computationally too expensive to run for 800 genes so no results are presented for this size for Genie3.

**Fig. 2.**
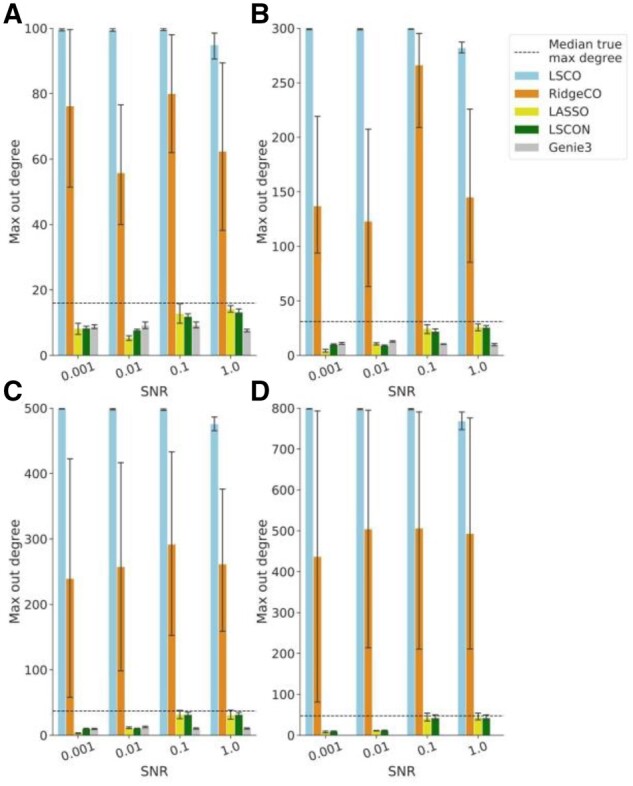
Maximum node degree for predicted GRNs of varying SNR on hub-prone data. Ten datasets were used for GRN inference with LSCON, LSCO, LASSO, RidgeCO and Genie3. The prediction was done on simulated data containing infinitesimal values and the network selected was the predicted GRN with a median degree closest to the median degree of the true GRN, from a set of 30 GRNs of varying sparsity generated for each dataset by each method. The simulated data contained 100 (**A**), 300 (**B**), 500 (**C**) or 800 (**D**) genes corresponding to the titles in the figure. The average maximum degree of the true GRNs is shown as a dotted line. The simulated data were generated from GRNs with scale-free topology

The first test evaluated, perhaps the one most aligned with the goal of LSCON, was the ability to avoid predicting megahubs. To ensure a fair comparison, all networks were selected with a median degree as close as possible to the true GRN. Here, LSCON, LASSO and Genie3 performed well, producing a maximum degree near or below the true maximum degree (Fig. 2). In comparison, LSCO and RidgeCO identified massive megahubs at all SNR levels. For all sizes the LSCO megahubs were connected to almost all other genes. The maximum degree in the GRNs for the other methods was almost always lower than in the true GRN. At the higher SNR levels, LSCON and LASSO GRNs were the closest to the true maximum degree.

**Fig. 3.**
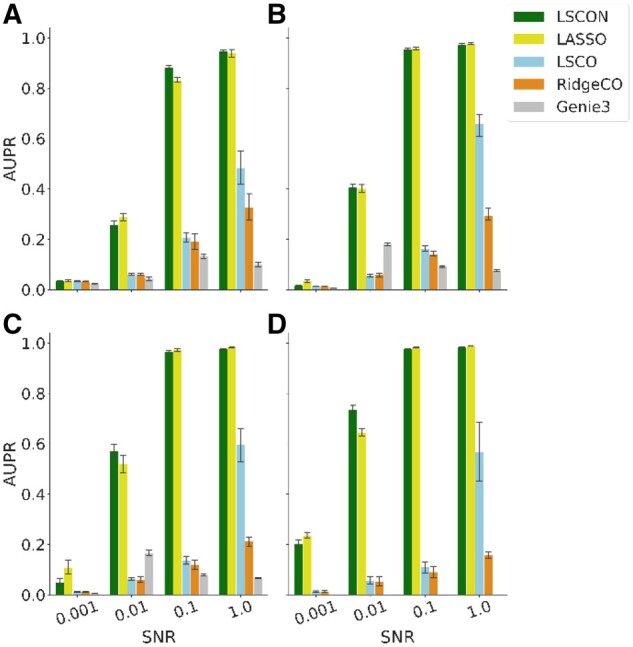
Accuracy of GRNs predicted from hub-prone data. LSCON, LSCO, LASSO, RidgeCO and Genie3 were used to predict GRNs from 10 datasets of 100 (**A**), 300 (**B**) and 500 (**C**) genes, as well as from 5 datasets of 800 (**D**) genes, corresponding to the titles in the figure. The simulated data contained small singular values and was generated from GRNs with scale-free topology. The AUPR is plotted over signal to noise ratio (SNR) as the methods are expected to perform better at higher SNR levels

Equally important to removing the amplification that causes megahubs is to ensure high accuracy of the predicted GRNs. To test for this the AUPR of the inferred GRNs was calculated on the simulated data for all methods, see Figure 3. For all sizes and noise levels, except SNR 0.001 where no method works, LSCON and LASSO perform about equally well and significantly outperform all other methods. They are followed by the considerably poorer performing LSCO, which in turn outperforms RidgeCO and Genie3 at SNR 1, but at other SNR levels all three methods perform poorly, with AUPRs below 0.2. For the three methods that avoided megahubs (LSCON, LASSO and Genie3), a tendency of increasing AUPR values was observed with increasing size of the input data, while for RidgeCO and LSCO the opposite trend was observed. As LSCON, LASSO and Genie3 do not find megahubs, they reach roughly the same AUPR as for data lacking infinitesimal values. We further note that when moving toward more biologically realistic noise levels (SNR 0.01), LSCON outperforms the LASSO method in the two larger datasets (*N* = 500 and *N* = 800; *P* = 0.15 and 0.007, respectively), adding support to the usefulness of our approach.

To examine the maximum accuracy achieved by each method, the F1-score metric was used. To compare the methods fairly we used the maximum F1-score across all sparsities. The findings further support the usage of LSCON for hub-prone data, with LSCON and LASSO again being the best performing methods with a maximum F1-score of between 0.8 and 0.9 for hub-prone data at SNR 1.0. LSCO scored at around 0.6, and RidgeCO performed far worse at between 0.2-0.5 in max F1 depending on the number of genes. Finally, Genie3 performed the worst at around 0.2 (Supplementary Fig. S2). These results largely mirror the AUPR results.

### 3.1 Additional datasets

To ensure that the observed effects were not an artifact of the data used, we tested the methods on data generated with GeneNetWeaver, an alternative data simulation tool to GeneSPIDER. For the GeneNetWeaver data all linear regression methods had a moderate performance (AUPR ∼ 0.5) while Genie3 performed noticeably worse in terms of AUPR (∼0.1) and similarly to the other methods in AUROC (Supplementary Fig. S3). Despite the high noise a similar trend as with the GeneSPIDER data can be observed, namely that LSCON performs equal to the other methods. Note that since the GeneNetWeaver data are not hub-prone, LASSO, LSCO, RidgeCO and LSCON perform about equally and are not significantly different (*P* = 1) as previously observed for balanced GeneSPIDER data.

**Fig. 4.**
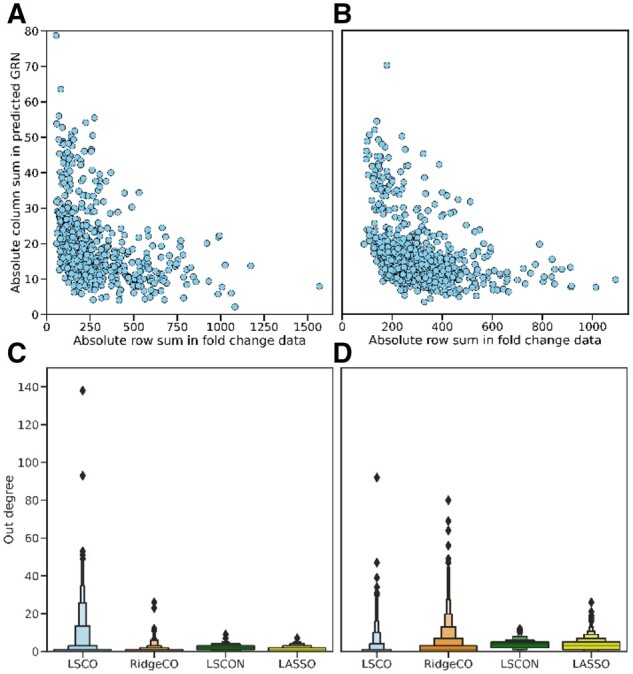
Analysis of GRNI with experimental data. (**A**) and (**B**) show the relationship between a gene's fold change in the input data and the magnitude of the predicted regulatory effect, predicted with LSCO. The data come from two cell lines A375 (A) and A549 (B). A clear trend is seen where genes with low-fold change tend to get a higher predicted regulatory effect compared with those genes with a high expression. Panels (**C**) (cell line A375) and (**D**) (cell line A549) show the degree distribution for four GRN inference methods: LASSO, LSCON, RidgeCO and LSCO, for one GRN per method which was selected by having the sparsity closest to three links/node

To further evaluate LSCON’s performance on real data, we carried out large-scale (∼600 to 700 genes) inference of GRNs for the cell lines A375 and A549 from the L1000 project. First, we demonstrated the megahub effect in these data by looking at the relationship between a gene’s fold change in the input data and the regulatory importance in the predicted network (Fig. 4A and B). This showed a clear trend for LSCO to give higher weights in the predicted network for genes with low fold changes and low weights for those with a high fold change. Next, we inspected the degree distribution of one GRN for each method, after selecting the GRN across all sparsities with an average node degree closest to three. LSCON results were compared with LSCO, RidgeCO and LASSO results, while Genie3 was not tested as it previously did not show a tendency of megahubs and has a prohibitive run time for data of this size. For these cell lines, the trend found in the simulated data is mirrored, with LSCO and RidgeCO finding large hubs of up to 140 edges, compared to the maximum of 15 edges for LSCON and 25 for LASSO (Fig. 4C and D). These results clearly demonstrate LSCON’s capability of avoiding megahubs for data where LSCO and RidgeCO would fall victim to the effects of infinitesimal values.

**Fig. 5.**
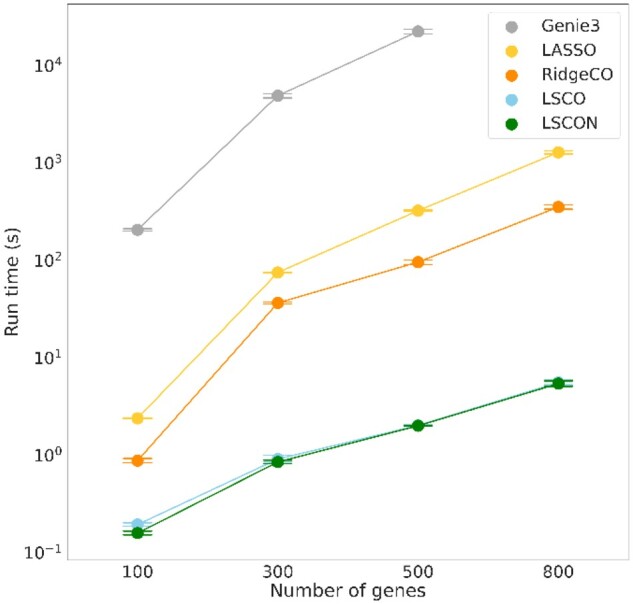
Mean execution time for five GRNI methods. The methods LSCO, LSCON, LASSO, RidgeCO and Genie3 were run on simulated data of varying sizes and their execution time measured in CPU time. Ten datasets at each size were used for determining average runtime for each method. Due to the excessive runtime of Genie3, we could not run it on the 800 gene datasets as the total runtime exceeded 100 wall clock hours

Finally, to ensure that the methods can be used for large-scale GRNI, either for inferring large GRNs or many GRNs in a bootstrapping setup, we compared their execution times. As LSCO and LSCON are based on the same algorithm, their speeds were essentially identical. With an execution time of <1 s for a 100 gene dataset to about 25 s on a 800 gene dataset they are both highly scalable and can easily be run in thousands of bootstrap iterations. In comparison, LASSO, and RidgeCO run about 10 times slower, and Genie3 had a runtime far greater than any of the other methods, and more than 1000 times slower than LSCON. For example, Genie3 required about 6 h for the 500 genes dataset, which LSCON runs in a few seconds (Fig. 5).

## 4 Discussion

The presented new GRNI algorithm, LSCON, was shown to consistently achieve top performance in both accuracy and speed, which is not accomplished by any of the other tested methods. This makes it an excellent choice for large-scale GRNI, both for tackling large numbers of genes, and many repeated inferences, e.g. during bootstrapping. LSCON performed similarly to the LASSO algorithm in correctness, while outperforming LSCO, RidgeCO and Genie3 on data with infinitesimal fold change values. LSCON was shown to perform identically to LSCO on balanced data, suggesting that it can replace LSCO in any situation.

We show that the data property which causes LSCO and RidgeCO to produce megahubs is found in biological data. This is further supported by previous work studying outlier detection and handling in both microarray (Kadota *et al.*, 2003; Shieh and Hung, 2009; Yang *et al.*, 2009) and sequencing data (Love *et al.*, 2014; Mangiola *et al.*, 2021). For GRNI, an example of a cause for such an outlier is the inclusion of a gene that is not part of the studied system. This gene would not change when the system is perturbed and hence all fold changes would become negligible. This is likely to be a common occurrence, and with an increasing number of studied genes in a study it will become even more common.

LSCON was found to be about 1000 times faster than Genie3, which is perhaps not surprising given that Genie3 generates 1000 trees to build its GRN from. Despite this heavy computation, Genie3 was outperformed by LSCON and LASSO in accuracy, even though these methods were used in single-run mode. With LSCON and LASSO run in single-run mode for this study, it is important to note the possibility to improve their accuracy by using bootstrapping techniques. A bootstrapping method that has previously been shown to work well with LSCO and LASSO is the Nestboot method (Morgan *et al.*, 2019). However, for infinitesimal data, the usage of LSCO is discouraged, and while LASSO can handle such data, it is generally too slow for large-scale applications. To make good use of bootstrapping, a complete inference should run 100 000 repeated GRN inferences of bootstrap samples (Morgan *et al.*, 2019). With LASSO this would take about 4 months for 500 genes, while LSCON would take about 3 days, making it a feasible task.

In addition to its excellent performance, LSCON also benefits from the inherited functionality with tools previously designed for LSCO thanks to the high similarity between the two in structure and use. LSCON is implemented in the GeneSPIDER package giving easy access to a large set of tools for simulation of GRNs and data, GRNI and analysis of data and predicted GRNs.

In conclusion, we have here shown that LSCON can significantly outperform the previously published LSCO method on hub-prone data and perform equally well for balanced data. This allows the LSCON method to be used in any place where the older LSCO method was previously used. As they are part of the same software package this can be done with little to no changes in existing pipelines as both in and output are identical between the tools allowing for simple updating of procedures and securing GRNI procedures from amplification and reduction issues.

## Funding

The authors thank the Swedish Strategic Research Foundation for financial support. This project was performed with grant VR 2019-04095.

*Conflict of Interest*: none declared. 

## Supplementary Material

btac103_supplementary_data
