# Supplementary material for: Fast and accurate gene regulatory network inference by normalized least squares regression
Source: Bioinformatics. 2022 Feb 17;38(8):2263–8. doi: 10.1093/bioinformatics/btac103 (PMC9004640; doi:10.1093/bioinformatics/btac103)
Supplement: btac103_supplementary_data [file btac103_supplementary_data.pdf]

Supplemental information for: Fast and accurate gene regulatory network inference by normalized least squares regression.

Thomas Hillerton[1], Deniz Seçilmiş[1], Sven Nelander[2], Erik L. L. Sonnhammer[1]*

[1]Department of Biochemistry and Biophysics, Stockholm University, Science for Life Laboratory, Box 1031, 17121 Solna, Sweden
[2]Science for Life Laboratory, Department of Immunology, Genetics and Pathology, Uppsala University, Uppsala, Sweden

*To whom correspondence should be addressed.

Supplemental note 1: Data generation

GeneSPIDER data generation

Simulated data allows for a greater variety in, and control of important data properties such as noise levels and condition number of the data. For this study, the simulated data consisted of 10 datasets for every combination of four different numbers of genes and four levels of signal to noise ratio (SNR). All datasets had a condition number less than 1000. The data was generated from simulated GRNs with a mean degree of 3 links per gene and a scale-free topology. The datasets were generated with SNRs 0.001, 0.01, 0.1 and 1, with 100, 300, 500 or 800 genes. The detailed properties of the data are available in Table 1, note that the properties are presented as the average over the 10 datasets to give a general view of the data. The properties of the networks are similarly available in Table S2. Note that the true GRNs are the same for different SNRs and tendencies to form hubs. All data generation was performed according to the original GeneSPIDER publication(Tjärnberg *et al.*, 2017).

Table S1: Average properties of the GeneSPIDER generated data

#datasets	Causes Megahubs	#genes	SNR	condition number	max	median	min	variance
10	No	100	0.001	3.58	14.39	-3.47E-03	-13.58	11.940
10	No	100	0.01	3.81	1.65	-5.12E-03	-2.00	0.136
10	No	100	0.1	10.43	0.96	-6.60E-04	-1.18	0.018
10	No	100	1	11.92	0.94	-1.17E-04	-1.13	0.017
10	Yes	100	0.001	3210.89	14.39	-2.92E-04	-13.56	10.792
10	Yes	100	0.01	19171.78	1.64	-3.34E-04	-2.00	0.123
10	Yes	100	0.1	9961.65	0.96	-3.53E-05	-1.18	0.016
10	Yes	100	1	18617.65	0.94	-9.04E-06	-1.13	0.015

10	No	300	0.001	3.65	4.75	-3.29E-03	-4.79	1.007
10	No	300	0.01	5.55	1.07	-3.23E-04	-1.35	0.015
10	No	300	0.1	13.43	0.94	-5.15E-05	-1.16	0.005
10	No	300	1	14.40	0.94	-6.90E-06	-1.15	0.005
10	Yes	300	0.001	11357.06	4.75	-2.70E-04	-4.78	0.958
10	Yes	300	0.01	16899.42	1.07	-3.42E-05	-1.35	0.014
10	Yes	300	0.1	53220.27	0.94	-4.47E-06	-1.16	0.005
10	Yes	300	1	12103.85	0.93	-6.79E-07	-1.15	0.005
10	No	500	0.001	3.68	2.74	-1.70E-03	-2.98	0.340
10	No	500	0.01	6.55	1.00	-1.75E-04	-1.24	0.006
10	No	500	0.1	14.07	0.92	-1.59E-05	-1.09	0.003
10	No	500	1	14.79	0.92	-2.39E-06	-1.08	0.003
10	Yes	500	0.001	47626.54	2.74	-1.03E-04	-2.98	0.324
10	Yes	500	0.01	38295.72	1.00	-1.28E-05	-1.24	0.006
10	Yes	500	0.1	52585.68	0.92	-1.29E-06	-1.09	0.003
10	Yes	500	1	65640.13	0.92	-1.74E-07	-1.08	0.003
5	No	800	0.001	3.69	1.80	-5.45E-04	-2.07	0.109
5	No	800	0.01	7.64	0.98	-7.12E-05	-1.11	0.003
5	No	800	0.1	15.36	0.94	-6.38E-06	-1.05	0.002
5	No	800	1	15.85	0.94	-1.70E-06	-1.05	0.002
5	Yes	800	0.001	76193.26	1.80	-1.17E-05	-2.07	0.104
5	Yes	800	0.01	93912.59	0.98	-3.92E-06	-1.11	0.003
5	Yes	800	0.1	147876.4	0.93	-2.33E-07	-1.05	0.002
5	Yes	800	1	226055.6	0.94	-9.83E-08	-1.05	0.002

Table S2: Average properties of the GeneSPIDER generated GRNs

Nr. of GRNs	Nr of nodes	Max in-degree	Max out-degree	Median degree	Median shortest path length
10	100	13.3	14.8	3.1	4
10	300	21.9	28.3	3	4.9
10	500	27.5	35.9	3	5
5	800	37	46.8	3	5

GeneWetWeaver data generation
In order to add a second source of synthetic data, we generated 5 networks using the GeneNetWeaver tool (Schaffter *et al.*, 2011) The networks were of size 100 with half of the genes assigned to regulators, random vertex seeding and neighbor selection from the top 50 genes was used to select the genes to include. From each network, 3 datasets were generated to serve as replicates of one single dataset using single knockdown perturbations with both ordinary differential equations (ODE) and a stochastic model (SDE). The noise coefficient of the stochastic model was set to 0.05, and measurement error modeled from noise in microarrays was added. The dataset was normalized separately after the merging, and not within the GeneNetWeaver tool. The normalization was performed by applying Z-score normalization for each column (experiment) in the data. To measure the difference in expression, log2 fold change was calculated by dividing the simulated experiment by its wild type profile, for both the noise-free data and noise itself. Once the fold changes were calculated for both, noise in fold change was added to the noise-free fold change. The settings were selected to mimic the properties of the DREAM4 data as closely as possible. The reason for not directly using the DREAM4 or DREAM5 (Schaffter *et al.*, 2011; Greenfield *et al.*, 2010; Marbach *et al.*, 2012) data was due to the lack of replicates that introduces potential limitations for several calculations in this study, including SNR. More detailed properties for both the data and the networks are presented in Table 3 and 4 respectively. Note that unlike the GeneSPIDER data in Table 1 and 2 these values are not averaged as only 5 datasets were generated and used with GeneNet Weaver.

Table S3: Data properties of the GeneNetWeaver data

#datasets	Causes Megahubs	#genes	SNR	Condition number	max	median	min	variance
1	No	100	1.17E-17	6.63	10.33	0.09	-13.84	2.01
1	No	100	1.32E-17	4.36	9.99	0.09	-13.36	2.01
1	No	100	1.73E-17	4.81	11.12	0.08	-12.96	2.00
1	No	100	2.00E-17	21.93	11.38	-0.06	-10.26	2.07
1	No	100	1.25E-17	4.77	12.72	0.07	-13.60	2.03

Table S4: Network properties of the GeneNetWeaver data

Nodes	Max in-degree	Max out-degree	Median degree	Median shortest path length
100	8	29	2	2
100	4	71	2	1
100	8	24	2	2

100	8	24	2	2
100	8	28	2	2

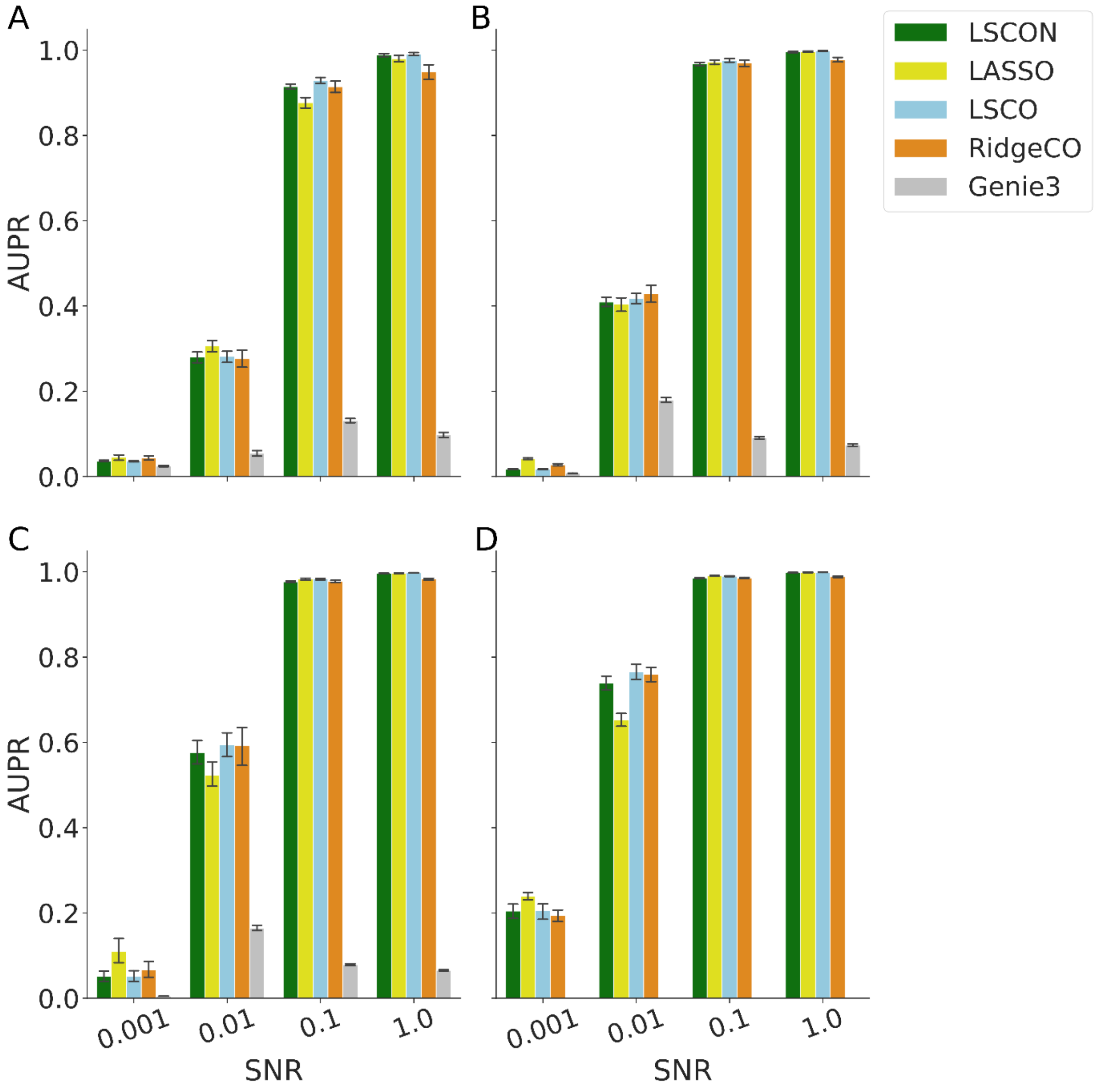

Supplementary figure 1: AUPR for data without infinitesimal values. The figure shows the AUPR for each SNR and each number of genes tested; 100 (A), 300 (B), 500 (C) and 800 (D). Note that due to the time complexity of Genie3 it was not possible to run it on 800 genes and thus no results are presented here.

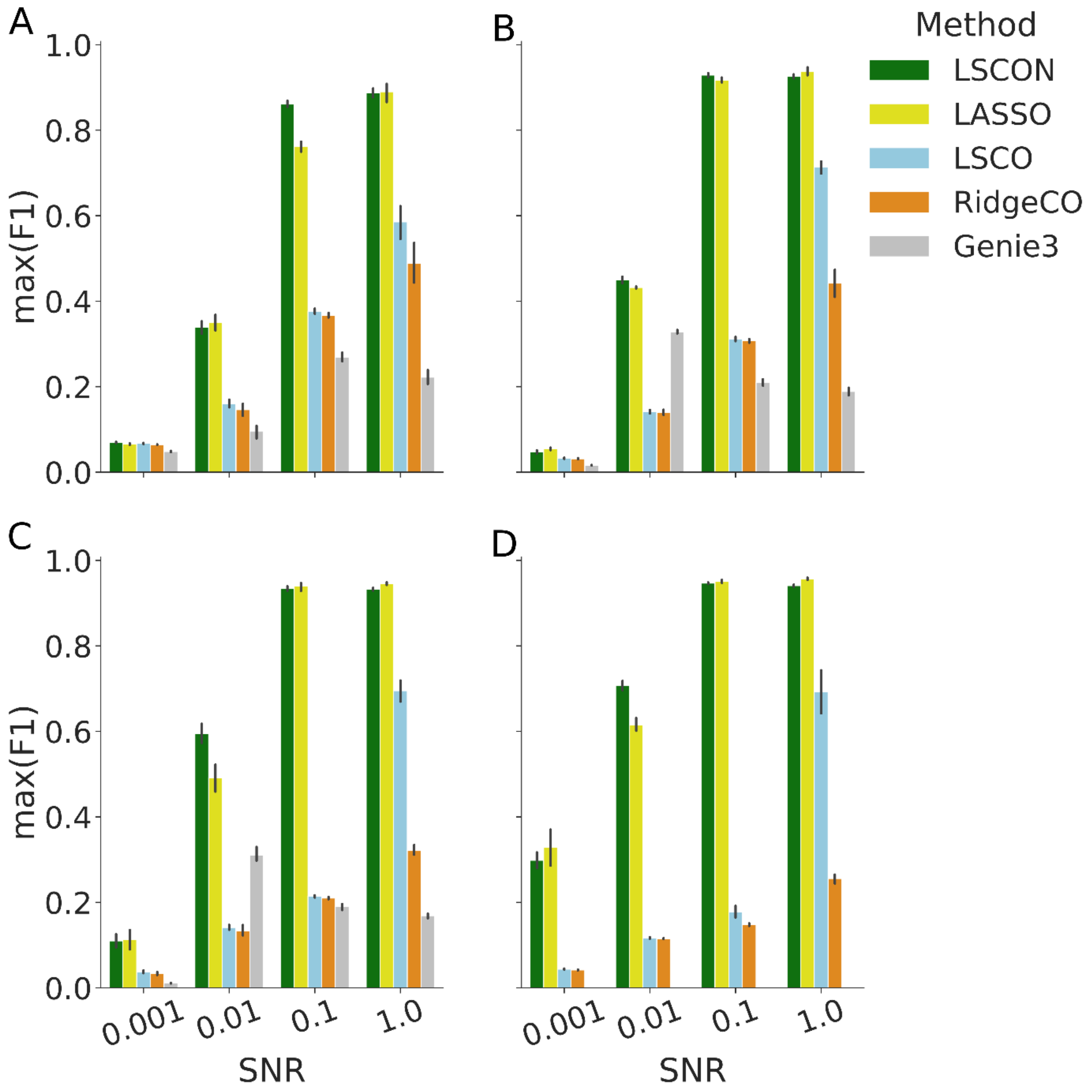

Supplemental figure 2: Maximum F1 score for predicted GRNs. When running the methods used in this study all methods produce GRNs at multiple sparsities, number of total edges. To evaluate the methods at their optimum, we here show the maximum F1 score obtained among all sparsities for multiple noise levels (SNR) and number of genes: 100 (A), 300 (B), 500 (C) and 800 (D).

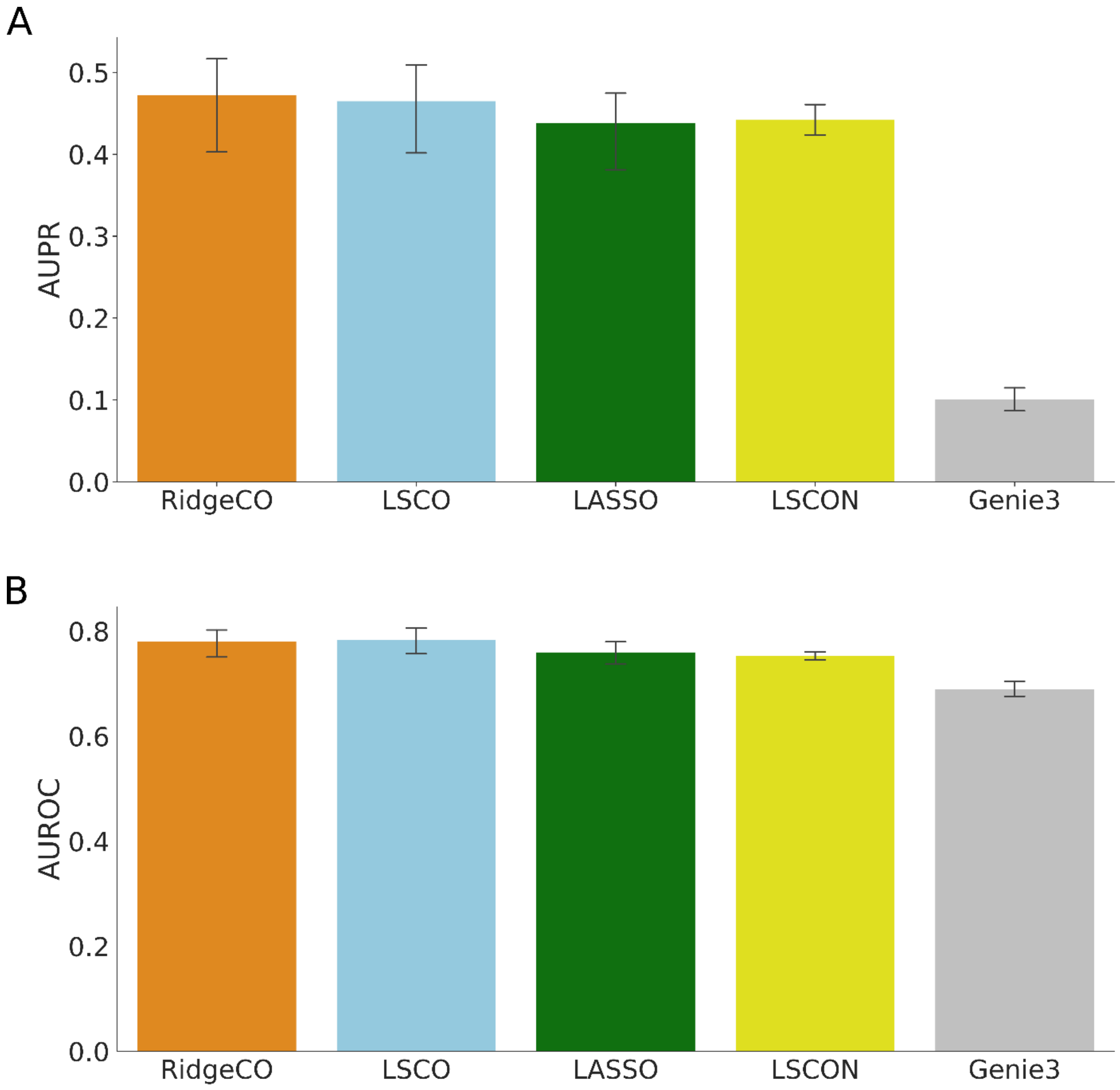

Supplementary figure 3: Correctness for GeneNetWeaver data. All methods were compared on GeneNetWeaver data in two categories AUPR (A) and AUROC (B). This data was here used to verify that the relatively good performance of LSCON was not due to some property of the GeneSPIDER simulation model.

References:

Greenfield,A. *et al.* (2010) DREAM4: Combining genetic and dynamic information to identify biological networks and dynamical models. *PLoS One*, **5**, e13397.

Marbach,D. *et al.* (2012) Wisdom of crowds for robust gene network inference. *Nat. Methods*, **9**, 796.

Schaffter,T. *et al.* (2011) GeneNetWeaver: in silico benchmark generation and performance profiling of network inference methods. *Bioinformatics*, **27**, 2263–2270.

Tjärnberg,A. *et al.* (2017) GeneSPIDER – gene regulatory network inference benchmarking with controlled network and data properties. *Molecular BioSystems*, **13**, 1304–1312.
